# Supplementary material for: Activity in serotonergic axons in visuomotor areas of cortex is modulated by the recent history of visuomotor coupling
Source: Peer Community J. Author manuscript; Available in PMC 2025 Aug 13. (PMC7618003; doi:10.24072/pcjournal.592)
Supplement: Supplementary Materials [file EMS207809-supplement-Supplementary_Materials.pdf]

Appendices

Table S1 - Statistical information on all analyses.

Figure panel	Value compared	Test	P-value	N (ROIs)	N (Sites)	N (Mice)
**1D**	Locomotion onset response in serotonergic axons in A24b	-	-	2697	11	4
**1E**	Grating onset response in serotonergic axons in A24b	-	-	1146	5	4
**1F**	Visuomotor mismatch response in serotonergic axons in A24b	-	-	1343	6	4
**1G**	Fraction of serotonergic axons in A24b responsive to locomotion onsets	Hierarchical bootstrap	$<10^{-4}$	-	**11**	4
	Fraction of serotonergic axons in A24b responsive to grating onsets	Hierarchical bootstrap	0.71	-	**5**	4
	Fraction of serotonergic axons in A24b responsive to mismatch onsets	Hierarchical bootstrap	0.59	-	**6**	4
	Fraction of serotonergic axons in A24b responsive to locomotion onsets *vs* grating onsets	Hierarchical bootstrap	$<10^{-4}$	-	**11,5**	4,4
	Fraction of serotonergic axons in A24b responsive to grating onsets *vs* mismatch onsets	Hierarchical bootstrap	0.75	-	**5,6**	4,4
	Fraction of serotonergic axons in A24b responsive to locomotion onsets *vs* mismatch onsets	Hierarchical bootstrap	$<10^{-4}$	-	**11,6**	4,4
**1H**	Locomotion onset response in serotonergic axons in V1	-	-	1812	10	4
**1I**	Grating onset response in serotonergic axons in V1	-	-	1050	5	4
**1J**	Visuomotor mismatch response in serotonergic axons in V1	-	-	1159	6	4
**1K**	Fraction of serotonergic axons in V1 responsive to locomotion onsets	Hierarchical bootstrap	$<10^{-4}$	-	**10**	4

	Fraction of serotonergic axons in V1 responsive to grating onsets	Hierarchical bootstrap	$<10^{-4}$	-	**5**	4
	Fraction of serotonergic axons in V1 responsive to mismatch onsets	Hierarchical bootstrap	0.88	-	**6**	4
	Fraction of serotonergic axons in V1 responsive to locomotion onsets *vs* grating onsets	Hierarchical bootstrap	$<10^{-4}$	-	**10,5**	4,4
	Fraction of serotonergic axons in V1 responsive to grating onsets *vs* mismatch onsets	Hierarchical bootstrap	$<10^{-4}$	-	**5,6**	4,4
	Fraction of serotonergic axons in V1 responsive to locomotion onsets *vs* mismatch onsets	Hierarchical bootstrap	$<10^{-4}$	-	**10,6**	4,4
**2B**	Correlation of serotonergic axon activity with locomotion velocity *vs* pupil diameter in A24b	Hierarchical bootstrap	0.005	**2697**	11	4
**2C**	Correlation of serotonergic axon activity with locomotion velocity *vs* pupil diameter in V1	Hierarchical bootstrap	0.03	**1812**	10	4
**3A**	Mean activity of serotonergic axons in A24b in closed loop *vs* open loop	Hierarchical bootstrap	0.18	**351**	3	3
**3B**	Mean activity of serotonergic axons in V1 in closed loop *vs* open loop	Hierarchical bootstrap	0.03	**471**	4	4
**4A**	Mean activity of serotonergic axons in A24b in closed loop under low *vs* high visuomotor gain	Hierarchical bootstrap	0.04	**678**	5	4
**4B**	Mean activity of serotonergic axons in V1 in closed loop under low *vs* high visuomotor gain	Hierarchical bootstrap	$<10^{-4}$	**232**	3	4
**S1A**	Locomotion onset response in serotonergic axons in A24b	-	-	2697	11	4
**S1B**	Locomotion onset response in serotonergic axons in V1	-	-	1812	10	4
**S2**	Difference in locomotion onset response to grating onset response in serotonergic axons in A24b *vs* V1	Hierarchical bootstrap	0.03	**1146,1050**	5,5	4,4

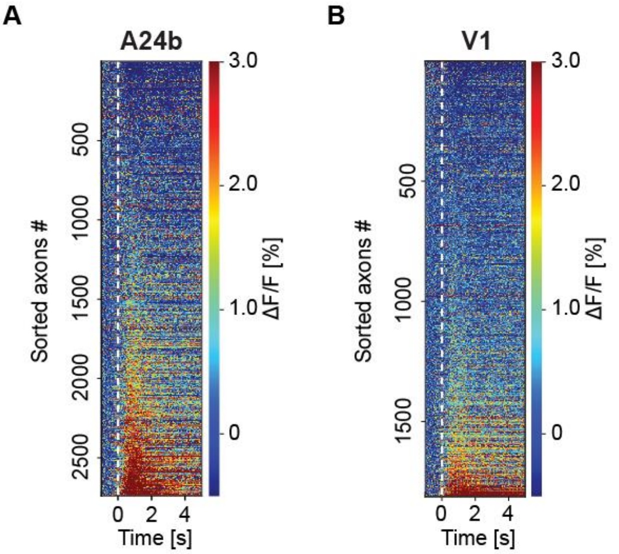

Figure S1 - Serotonergic axons were active on locomotion onset. (A) Average locomotion onset activity of all serotonergic axons in A24b, sorted by their average response during locomotion onset. Please note, shown are all data and sorting introduces regression to the mean artefacts, like the apparent decrease in a subset of axons on running onset. (B) As in A, but for serotonergic axons in V1.

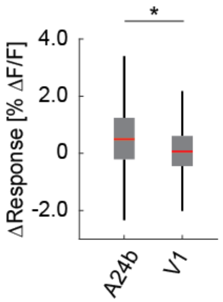

Figure S2 - Responses of serotonergic axons were area specific. The difference in the average response on locomotion onset to grating onset for individual serotonergic axons in A24b was higher than in V1. Boxes show 25th and 75th percentiles, central mark is the median, and the whiskers extend to the most extreme data points not considered outliers. n.s.: not significant; *p<0.05; **p<0.01; ***p<0.001; see Table S1 for statistical information.
